# STRENGTHENING OLDER INDIGENOUS NEW ZEALANDERS AT END OF LIFE: WHAT ROLE DO HEALTH SERVICES PLAY?

**DOI:** 10.1093/geroni/igz038.2470

**Published:** 2019-11-08

**Authors:** Tess H Moeke-Maxwell, Kathleen R Mason, Merryn Gott

**Affiliations:** University of Auckland, Auckland, New Zealand

## Abstract

Older indigenous people and their families draw on specific tribal care customs to support end-of-life care as these help to fortify and strengthen older people. New Zealand’s health and palliative care services can either help or hinder families to utilise their care customs. The aim of the Pae Herenga study was to investigate the specific traditional care customs employed by older New Zealand Māori. This involved 60 face-to-face interviews with participants who had a life limiting illness (majority aged over 65), family carers, indigenous healers, spiritual practitioners, and health and palliative care professionals across four key geographical sites. Three digital story workshops involving 16 participants were also included. The study findings show that no matter what the older person’s illness was, their cultural customs and protocols helped to fortify them and kept them spiritually safe at end-of-life. Hospitals and hospices helped families to action their customs by providing rooms large enough to host gatherings of thirty or more people; prayers, songs, speechmaking and communal sharing of food took place. However, incidences of racism, a lack of space, and a lack of support for indigenous plant medicines prevented the use of ancient traditional end-of-life care customs for older people. The findings suggest that health and palliative care services can help older indigenous people maintain their spiritual strength by providing them with culturally supportive care and environments equipped to host the dying and their families.

